# Spondyloarthritis in African Populations Compared to Europeans Living in the United Kingdom

**DOI:** 10.31138/mjr.32.1.39

**Published:** 2020-10-19

**Authors:** Euthalia Roussou

**Affiliations:** Rheumatology Department, Department of Rheumatology and Rehabilitation, Barley Lanes, United Kingdom

**Keywords:** Spondylarthritis, Africans, Europeans, United Kingdom, NHS, comparison

## Abstract

**Background:**

With the aim to study Spondyloarthritis in patients originating from Africa and compare the disease with the way it is manifested in Europeans, data was analysed from 62 African patients and compared with 56 Europeans living in the same geographical area (north East London, United Kingdom) and treated under the same health system (NHS). Data analysed were demographic, social and clinical characteristics.

**Results:**

Comparisons showed differences in prevalence of psoriasis (more in Caucasians), uveitis (more in Africans), smoking (more in Europeans), and significantly fewer patients of African origin declared family history of SpA. African patients have less disease activity (but not significantly better measured by BASDAI), and statistically significant better functional ability (BASFI) compared to Europeans. No difference has been noted in gender distribution, age of disease onset, disease duration, delay in diagnosis, disease associations with IBD, night pain, or overall wellbeing.

**Conclusions:**

SpA is different in Africans in that it shows to be milder in terms of disease activity and functional ability with more uveitis less psoriasis and less family history of SpAs.

## INTRODUCTION

Spondyloarthritis (SpA) comprises a group of inflammatory chronic diseases that share similar clinical presentations, radiological findings, human leucocyte antigen (HLA) B27 association and positive family history.^1^ According to the European Spondyloarthropathy Study Group (ESSG) criteria,^2^ SpA has been traditionally classified as: ankylosing spondylitis (AS), reactive arthritis (ReA), undifferentiated SpA (uSpA), psoriatic arthritis, and arthritis associated with inflammatory bowel disease (IBD).^3^ Its prevalence varies from 0.1% to 1.4% of the general population depending on the geographical region studied.^4^ Despite the fact that SpA affects males more frequently and severely than females, there is an increasing notion that the proportion of female patients is higher than initially thought, with women having less severe clinical manifestations.^5^ SpA usually begins in individuals younger than 45 years of age affecting the spine, peripheral joints, tendons and ligament insertions (entheses). It has extra-articular manifestations from the skin, mucosa, gut, and the eyes.^6^ HLA-B27 is considered a risk factor for the development of SpA. Although it is associated with AS (in particular), the association is variable across ethnic groups.^7^

The aim of the present study is to assess SpA in Africans compared to Europeans.

## PATIENTS AND METHODS

### Patients

Patients with any form of SpA who were evaluated by a rheumatologist in the outpatient department enrolled in a clinical registry of spondyloarthritis (SpA) called London Registry of SpA (LoRoS). Our group of hospitals has a catchment area of 700,000 inhabitants with varied ethnicities. Most of the data were derived from King George Hospital (Ilford, Essex), which predominantly serves the area of Redbridge, a region with marked ethnic variation.^8^ Data presented in this paper were obtained from questionnaires administered to patients attending their routine outpatient clinic for rheumatology or a combined rheumatology/dermatology clinic.

The patients were referred to our services by local general practitioners (GPs), consultants from other specialties within the hospitals, and by the local Musculoskeletal Clinical Assessment and Treatment (MCAT) services for back pain. These services are run by experienced physiotherapists who mainly treat mechanical back pain. Individuals with evidence of inflammatory back pain (IBP) or back pain with evidence of peripheral joint disease are flagged and referred for hospital assessment and treatment.

The baseline assessment of all referred patients included review of their medical history and clinical examination. Medical history included any family history of psoriasis, inflammatory bowel diseases, and other conditions associated with SpA, such as uveitis. Particularly attention has been given to those with history suggestive of IBP, as recommended by Calin^9^ and the Assessment of SpondyloArthritis criteria proposed by Rudwaleit for axial^10^ or peripheral SpA.^11^ Laboratory tests are taken place routinely and include erythrocyte sedimentation rate (ESR) and C-reactive protein (CRP) for all patients with evidence of Inflammatory arthritis.

Radiological investigations included radiographs of the hands, wrists, feet, and ankles if there was evidence of peripheral disease, in addition to radiographs of the spine (cervical, thoracic, and lumbar spine), the hips, and pelvis. If sacroiliitis could not be clearly confirmed from radiographs of the hips and pelvis, (a plain anterior pelvic X-ray and a dedicated X-ray of the sacroiliac joints) a magnetic resonance imaging (MRI), usually with contrast enhancement, of the sacroiliac joints was requested as recommended before.^12^ Patients with confirmed SpA completed a semi-structured questionnaire, as described below.

### Questionnaire

The semi-structured questionnaire was originally developed in 2001 and was validated and used in this group of patients in 2004. The data presented here were collected between 2005 and 2016.

Patients were recruited when attending the outpatient clinics of our hospital. On attendance, patients with evidence of IBP and confirmed diagnosis of SpA were given the 11-page questionnaire together with an information sheet explaining the aims of the study and a consent form. The patient’s questionnaire contained seven groups of questions, namely demographic data, disease onset, first symptom at disease onset, and main symptoms experienced during the disease course. Age at diagnosis, disease duration, and delay in diagnosis were obtained from the above-mentioned sections of the questionnaire. Two questions were asked about disease onset: “*When do you think the disease started?*”, and “*When was the disease diagnosed?”* By comparing the two responses, we could determine whether there was a delay in diagnosis.

The Bath Ankylosing Spondylitis Disease Activity Index (BASDAI)^13^ and the Bath Ankylosing Spondylitis Function Index [BASFI]^14^ were both included in the questionnaire to assess disease activity and functional ability, respectively. Disease activity was also assessed in terms of ESR and CRP. Two 10 cm visual analogue scales (VAS) were also included and asked patients to state their perception of well-being and night pain over the preceding week. In both VASs, 10 cm was defined as the worst possible response. In the demographics section, the patients were asked “*What is the ethnic group to which you belong*?” The patients chose from five lists: Asian (Bangladeshi, Chinese, Indian, Pakistani, or other), Black (African, Afro-Caribbean, or other), European (white British, or other), mixed (describe), and other (describe). For mixed and other, the patients were able to describe their exact ethnicity if it was not stated in the provided lists. Individuals of mixed ethnicity were excluded from this study.

Also, a question was included asking whether they were known to have osteoporosis and whether they ever had a DEXA scan.

The level of education they had reached, marital status and number of children was also obtained. Finally, there was a question on family history of spondyloarthritis and the degree of the relationship as described before.^17^

## VALIDATION OF THE QUESTIONNAIRE

During the validation process, the first 50 patients filling in data were questioned to confirm their age, gender, and race. The patients’ race was determined from the questions covering ethnicity and was verified by the physician. The symptoms at disease onset, clinical aspects, clinical association, employment status and family history were also tested. The symptoms at disease onset were validated with the symptoms described in the general practitioner’s referral letter.

In order to validate the clinical aspects, the patient’s clinician (assessor) also completed a two-page questionnaire to record clinical data such as joints with arthritis, symmetrical or asymmetrical pattern, whether they had confirmed sacroiliitis, documented inflammatory bowel disease, uveitis, previous infection (salmonella, Shigella, Yersinia, Chlamydia) associated psoriasis, enthesitis, or Juvenile onset disease. The clinician’s data were then examined against the reported patient’s data from the questionnaire. There are however questions on the questionnaire such as “*which is the **main** symptom(s) at disease onset and **main** problem(s) that the disease is causing to you*” which are based on perception and cannot be validated by a third party.

### Statistical analysis

Data are expressed as mean ± SD (except where otherwise indicated). Statistical analysis performed using *χ^2^* tests for comparisons between categorical variables (ie, sex and symptoms) and independent sample t-test was used to assess differences between means of two sets. Two-tailed p values <0.05 were considered statistically significant. For statistical analysis we used SPSS V.19.0 (SPSS Inc). For the presentation and analysis of categorical variables, frequency distribution, percentages and contingency tables were used.

### Ethical approval

The study was approved by the Redbridge and Waltham Forest Ethics Committee and by the Research and Development Board of Barking Havering and Redbridge University Hospitals NHS Trust. Each patient signed an informed consent form and confidentiality was strictly maintained.

## RESULTS

From a total of 776 patients that provided data and were included in a registry of spondyloarthropathies, 62 patients originating from Africa (and living in the UK) with a diagnosis of SpA according to the ESSG criteria were studied. This suggests a prevalence of 7.9% of SpA in African individuals. Their data were compared with 56 patients who had declared themselves as Europeans. All of the patients were seen at King George Hospital in North East London (Redbridge), UK, between January 2005 and December 2016. All patients were evaluated by the same rheumatologist in the outpatient department. **Table 1** shows demographic, clinical, and social characteristics of the 2 groups studied, as well as differences between the 2 groups.

**Table 1.** Demographic, clinical, and social characteristics of the 2 groups studied.

**Item**	**African (N=62)** **DIAGNOSES** **AS = 15 (24.1%)** **PsA= 11 (17.7 %)** **ReA= 3 (4.8 %)** **USpA= 20 (32.2%)** **UC= 2 (3.2%)** **ACHILLES TENDINOSIS = 1 (3.2%)**	**European (N=56)** **11 (17.5%)** **22 (34.6%)** **1 (1.6%)** **9 (13.3%)** **1 (1.6%)** **0**	**ss**	**Comment**
M/F	22/40	20/36	0.38	NS

Age (m + sd)	44.6 +12.4	46.4 + 13.6	0.5	NS

ESR	18.6 + 18.3	21.1 + 22	0.6	NS

AGE diagnosis	39 + 13.3	39.7 + 14	0.6	NS

Disease duration	10 + 9.9	11.6 + 11.1	0.9	NS

Delay in diagnosis	6.4 + 7.08	6.5 + 7.9	0.5	NS

HLA B27 _+_	2	6 (9.5%)	0.9	NS

SMOKING (yes)	7 (11%)	18/53 (34%)	0.001	significant

Alcohol (yes)	28 (47.5%)	37/53 (69.8%)	0.68	NS

Eye inflammation (uveitis)	14 (28.6%)	6 (11.3)	0.2	NS

Psoriasis	6 (12.8%)	16 (34%)	0.03	significant

Irritable bowel syndrome	15 (23.6%)	19 (38%)	0.3	NS

BASDAI	6.2 + 2.1	6.6. + 1.6	0.3	NS

BASFI	4.8 + 2.7	5.9 + 2.4	0.03	significant

NIGHT pain	5.8 + 3.04	6.3 + 3.3	0.5	NS

Effect of treatment (VAS)	3.8 + 2.8	3.2 + 2.8	0.1	NS

WELL being (w)	6.4 + 2.5	6 .03 + 3.01	0.2	NS

Osteoporosis	6 (10) 10%	5 (9.4%)	0.4	NS
DEXA	7 (12.5%)	9 (16.1%)	0.6	NS

Education			0.02	
0	3 (5.5%)	4 (8%)		CI
1	4 (7.3 %)	6 (12%)		−0.098 to −1.251
2	9 (16.4%)	14 (28%)		
3	13 (23.6 %)	14 (28%)		
4	23 (41.8 %)	12 (24%)		
5	3 (5.5%)			

Marital status			0.7	CI
1	33 (55.9%)	32 (61%)		−.0512 to 0.349
2	20 (33.9%)	10 (19.2%)		
3	3 (5.1%)	4 (7.7%)		
4	2 (3.4%)	4 (7.7%)		
5	1 (1.7%)	2 (3.8%)		

Children (number of)			0.4	CI
0				−0.443 to 1.021
1	13 (22.8)	8 (16 %)		
2	3 (5.3)	4(8%)		
3	15 (26.3)	13 (26 %)		
4	12 (21.1)	12 (24%)		
5	7(12.3)	8 (16 %)		
6	1 (1.8)	3 (6%)		
7	5 (8.8)	2 (4%)		
8	0			
9	0			
	1 (1.8%)			

FX (yes)	19/52 (36.5%)	33/53 (63.5%)	0.02	CI
				−0.503 to −0.042

## DISCUSSION

Our study represents a retrospective analysis of data from patients with SpA living in a defined catchment area in north East London. In this study, although the registry had 776 patients at the time of the analysis, in order to avoid bias we excluded patients declaring themselves as “British”, as British identity does not distinguish race and data. Data only from patients with clear origin were used and analysed. Such a choice provided limitations in reaching statistical analysis; therefore, these results may represent initial results requiring further testing.

Through the study, differences have been identified in SpAs between African and European populations living in the same multi-ethnic geographical area, exposed to the same environmental (weather, food, pollution) conditions, and treated under the same health system. Results showed that although African patients with SpA have comparable gender distribution, age at disease onset, disease duration, delay in diagnosis, and clinical characteristics such as association with irritable bowel, disease activity, night pain, effect of treatment, and overall wellbeing with Europeans, they also have differences all indicative of milder disease expression in Africans. These differences are related predominantly to genetic background (defined in our study by family history), social habits (smoking), prevalence of psoriasis and functional ability. A clearly distinct prevalent association in the African population is that of increased prevalence of uveitis (29%) compared to the prevalence seen in Europeans (11%), which, however, has not reached statistical significance levels.

With regards to genetic background, the answer to the Family history question indicates that more Europeans have reported positive family history of SpAs compared to African patients. HLA-B27 has been known for over 40 years as a predisposing factor for SpA, specifically for AS.^15^ In Caucasians with AS, 80–85% of patients are HLA-B27 positive. Overall, however, a small percentage of Caucasian individuals who are HLAB27 positive will subsequently develop the disease.^16^ It seems that in populations other than Caucasian, HLA-B27 is less prevalent. Since it is known that the HLAB27 allele is virtually absent in Africa, it can perhaps be suggested that the genetic predisposition is HLAB27-related, and in its absence, there is no family aggregation or family predisposition.

Another interesting finding is that there is greater incidence of Psoriasis in Europeans. Our study shows that 12.8% of SpA patients of African origin have psoriasis compared to 34% of the Europeans. This is comparable with data published in a paper from South Africa^18^ in which in 2003 it was reported that from just over 7000 patients assessed of whom 76% were black, 10% white, and 7.6% were Indian, eczema was the most common dermatological disorder identified in the black population (37%) followed by acne (17.5%). In South Africa psoriasis was seen in a relatively small proportion (17.5%). This was from those of Asian descents rather than native African descent.

Our patients are mostly from Nigeria, and to generalize for the whole of the African continent would perhaps indicate it as an overstatement. However, by taking together these data from South Africa, and our small sample from African patients who are predominantly of Nigerian origin (representative of another African country not neighbouring to south Africa), one can assume that perhaps Africans as a whole have less psoriasis. More data are needed in the prevalence of psoriasis and psoriatic arthritis in the African continent.

Africans have better functional ability (defined by BASFI) compared to Europeans, but also better disease activity as defined by BASDAI. Smoking has been associated with increased disease activity.^19^ Although BASDAI was elevated in Europeans a greater percentage of whom are smokers, compared to African this difference in disease activity did not reach significance levels. This may be related to the small number of patients examined. The difference in the functional ability however between Africans and Europeans was significant indicating that African patients run milder disease. Since we have identified increased proportion of patients with uveitis, one may suggest that SpA in African populations is different with more systemic manifestations such that the one from the eyes rather than the musculoskeletal system.

The notion that the SpA spectrum of diseases are not common in Africa has been suggested for many years. There are reports from South Africa stating that Spondyloarthritis has similar characteristics in South Africa as in Europe since the late 1990s and early 2000s.^20–21^ The fact that there has been more reactive arthritis in Africa following the spread of HIV/AIDS has not been seen in great proportion among the Africans migrating to Europe, and there was not anyone among our patients studied, although we have seen such patients in our department.

In summary, our data suggest that African patients with spondyloarthritis in addition to less genetic predisposition manifested by low incidence of HLAB27 have milder disease compared to Europeans with less psoriasis and more uveitis.

## Figures and Tables

**Table:** 

ASIAN	BLACK	EUROPEAN	MIXED	OTHER
Bangladeshi	African	Describe		Describe
Chinese	Caribbean		________	________
Indian	Other______	______		
Pakistani				
Other-------				

**Table:** 

Never smoked	
Ex-smoker	
Smoker	
Between 1–5 cigarettes/day	
5–10	
10–20	
More than 20	

**Table:** 

Never	
Daily	
Socially	

**Table:** 

Back pain	
Neck pain	
Knee pain/swelling	
Shoulder pain	
Foot pain/swelling	
Hip pain	
Eye inflammation	
Headache	
Don’t remember	
Other	

**Table:** 

Tiredness (Fatigue)	
Neck pain	
Upper back pain	
Lower back pain	
Stiffness	
Joint pain / swelling	
Pain with pressure on various areas	
Other (what)	

**Table:** 

Heart	
Lungs	
Dizziness	
Headaches	
Numbness	
Kidneys/ water works	
Other	
No other problems	

**Table:** 

Eye inflammation	
Psoriasis	
Dry skin in your hair or itchiness	
Dry skin elsewhere in body	
Irritable bowel	
Stomach irritation of any sort	

**Table:** 

0	1/2	1	11/2	2 or more
	hrs			hrs

**Table:** 

Pain killers	
Non-steroidal anti-inflammatory drugs	
Steroids	
Sulphasalazine	
Methotrexate	
Leflunomide	
Cyclosporine	
Anti TNF	

**Table:** 

a) Are you a vegan? (eating plant products ONLY)	Yes / No
b) Are you vegetarian?	Yes / No
If YES, do you eat any of the following?	
Eating fish	Yes / No
Eating chicken	Yes/No
Eating eggs	Yes/No
Dairy products	Yes/No
c) Do you eat fish or fish produce (ie fish oils) at least once a week	Yes/No
d) Do you think any foods aggravate your joint disease?	Yes/No

**Table:** 

Meat produce
Dairy produce
Lentils/Pulses/beans
Fruits
Alcohol
Fish
Carbohydrates (ie rice, potato, bread)
Other

**Table:** 

No school
Primary school
Secondary school
College or technical skills
Higher education

**Table:** 

Manual	
Active (moving)	
Sedentary (sitting)	
Housewife	
Student / artist	

**Table:** 

Unemployed and intending to work
Retired
Unemployed and unable to work

**Table:** 

Solely because of your disease?
Partly because of your disease?
For reasons other than your disease?

**Table:** 

1a) Have you ever seen a rheumatologist (a specialist in arthritis)?	YES/NO
1b) Have you ever been tested for HLA-B27 (a gene usually associated with ankylosing spondylitis)?	YES/NO
1c) If yes, was the result:	

**Table:** 

Positive	
Negative	
I don’t know	

**Table:** 

GP	
Rheumatologist	
Eye specialist	
No doctor	

**Table:** 

GP	
Rheumatologist	
Skin specialist	
No doctor	

**Table:** 

GP	(a)	(b)
Rheumatologist		
Skin specialist		
No doctor		

**Table:** 

a) Are you married	single	divorced	live with partner	widowed
□	□	□	□	□

**Table:** 

b) Were your parents blood related in any way prior to marriage?	yes	no
	□	□

## Notes

***Ref.no*** (for office use)

## LoRoS Questionnaire *(version 5)*

Spondyloarthropathies in multicultural populations in London.

Do you need an interpreter to fill in this questionnaire?  Yes □  No  □

1. *Demographics
*

FULL NAME: __________DATE (today): __________

ADDRESS __________ SEX: __________MARITAL STATUS: __________

DATE OF BIRTH: __________ Place of birth __________

If born outside UK when (year) did you arrive in the UK __________

E-MAIL ADDRESS (if there is any): ____________________

NAME OF Rheumatologist: _______________________________________

Contact OF DOCTOR (e-mail address/ postal or telephone): ____________________

To which of the following groups do you consider that you belong?

What is the main language spoken in your household? ____________________

**
SMOKING
**

**
DO YOU DRINK ALCOHOL?
**

**
2. Clinical I (Disease onset)
**

a)    When did your disease begin? (Year and age) ____________

b)    When was the diagnosis made? (Year and age) ________________

c)    What was your first complaint/symptom? Buttock pain

**
3. In your opinion, what is the main problems that the disease is causing to you? (** ***Please put a number in each box in order, 1 being worst, 2 being second worst, etc.*** **)
**

**
Clinical II (associations)
**

4) Other than joints/back pain, do you have any other problems with any of the following: *(tick more than one if there many)
*

**
5. Have you ever had OR now have: (** ***Tick more than one if there are many*** **)
**

**
6. Osteoporosis:
**

a) Have you been told that you have “brittle” bones/osteoporosis?  Yes  □  No  □

( *Tick the right one for you )
*

b) Have you ever had the special scan (DEXA)to confirm this?    Yes □  No  □

**
Clinical III (BASDAI)
**

7. PLEASE PLACE a vertical mark (for example____/_____) on each line below indicating your answer to each question (ranking your symptoms) relating to the PAST WEEK.

1) How would you describe the overall level of fatigue / tiredness you have experienced?

NONE _______________________________________________ VERY SEVERE

2) How would you describe the overall level of AS neck, back or hip pain you have had?

NONE _____________________________ VERY SEVERE

3) How would you describe the overall level of pain/swelling in joints other than neck, back or hips you have had?

NONE _____________________________ VERY SEVERE

4) How would you describe the overall level of discomfort you have had from any areas tender to touch or pressure?

NONE _____________________________ VERY SEVERE

5) How would you describe the overall level of morning stiffness you have had from the time you wake up?

NONE _____________________________ VERY SEVERE

6) How long does your morning stiffness last from the time you wake up in the past week?

**
a)** How would you describe the overall level of night pain you have had in the past week?

NONE __________________________________VERY SEVERE

**
b)** How would you describe the overall level of disturbance to your sleep due to your disease?

NONE __________________________________VERY SEVERE

**
8. Function:** Please place a mark on each line below to indicate your level of ability with EACH OF THE FOLLOWING ACTIVITIES during PAST WEEK: ( *N.B. An aid is a piece of equipment which helps you to perform an action or movement )
*

1) Putting on your socks or tights without help or aids (eg, sock aid)

EASY_______________________________________________ IMPOSSIBLE

2) Bending forward from the waist to pick up a pen from the floor without an aid

EASY_______________________________________________ IMPOSSIBLE

3) Reaching up to a high shelf without help or aids (eg, helping hand)

EASY_______________________________________________ IMPOSSIBLE

4) Getting up out of an armless dining room chair without using your hands or any other help

EASY_______________________________________________ IMPOSSIBLE

5) Getting up off the floor without help from lying on your back

EASY_______________________________________________ IMPOSSIBLE

6) Standing unsupported for 10 minutes without discomfort

EASY_______________________________________________ IMPOSSIBLE

7) Climbing up 12 - 15 steps without using a handrail or walking aid. One foot on each step

EASY_______________________________________________ IMPOSSIBLE

8) Looking over your shoulder without turning your body

EASY_______________________________________________ IMPOSSIBLE

9) Doing physically demanding activities (eg, physiotherapy exercises, gardening, or sports)

EASY_______________________________________________ IMPOSSIBLE

10) Doing a full day’s activities whether it be at home or at work

EASY_______________________________________________ IMPOSSIBLE

**
9. Surgery
**

Have you ever had surgery in any of your joints?    yes  □  no  □

If yes, what surgery (in which part of your body)

______________________________________________________________

**
10. Treatment
**

a) Are you currently taking any medication for your disease?     yes  □  no  □

If “YES” are you taking

b) Place a vertical mark (eg, ___/____) on the scale below to indicate the effectiveness that treatment has on relieving your symptoms:

NONE __________________________________________________VERY EFFECTIVE

**
11. WELLBEING
**

a)    Place a vertical mark on the scale below to indicate the effect your disease has had on your well-being over the last week:

NONE __________________________________________________WORST POSSIBLE

b)    Place a vertical mark on the scale below to indicate the effect your disease has had on your well-being over the last six months:

NONE _________________________________________________ WORST POSSIBLE

**
12. Dietary habits (** ***Please delete appropriately or circle the right one for you*** **)
**

If Yes, any of the foods below:

e) Do you think that any food helps your disease?      Yes/No

If yes, what

________________________________________________________________________________________

**
13. Education/ Occupation/Employment
**

a) What level of education have you reached? ( *Please tick each relevant box* )

b) Are you currently employed? (incl. self-employed) YES / NO

c) Is your occupation:

d) If not employed, are you

Are you UNEMPLOYED / RETIRED:

**
14. Spondyloarthropathy Group
**

**
2. IRITIS / UVEITIS
**

a) Do you get iritis (uveitis)?     YES/NO

b) If YES, who made the diagnosis?

c) Have you had any treatment for it?    YES/NO

    Please specify: ___________________________________________

d) When did your first symptom of iritis start?

  Year:    Age:

e) Has the uveitis resulted in persistent deterioration of vision?     YES/NO

**
3. PSORIASIS
**

a)    Do you get psoriasis?     YES/NO

b)    If YES, who made the diagnosis?

c) Have you ever had any treatment for it?     YES/NO

    Please specify (what): ____________________________

d) When did your first symptom of psoriasis start?

  Year:  Age:

e) Have you ever had sausage-like fingers  YES/NO

f) Do you have (or ever had) funny nails?  YES/NO

**
4. ENTEROPATHIC SPONDYLOARTHROPATHIES
**

1. Have you ever been diagnosed as having:

    a) Crohn’s disease     YES/NO

    b) Ulcerative colitis     YES/NO

2.    If YES, who made the diagnosis?

3.    When did your first symptom of bowel disease start?

  Year:  Age:

**
15. Family HISTORY
**

c) If yes what was the relationship prior to marriage? ________________

d) Do you have any children? Y □ N □ How many □

If yes

e) Please put down ages and gender (M=male or F=female) of children.

Age _______ Sex______ Age ______ Sex ______ Age ______ Sex ______

Age _______ Sex ______ Age ______Sex _______ Age ______Sex ______

f) Do you have any brothers or sisters? Y □ N □

If yes, please put down their ages and gender

Age _______ Sex______ Age ______ Sex ______ Age ______ Sex ______

Age _______ Sex ______ Age ______Sex _______ Age ______Sex ______

g) As far as you know do any other members of your family have any form of spondyloarthropathy, like:

Ankylosing spondylitis, Psoriatic arthritis, reactive arthritis, ulcerative colitis, or Crohn’s disease?

    Yes □ N □

Does anybody in your family suffer with **psoriasis** (flaky skin)?   YES/NO

If yes, what is the relationship to you? **_______________________
**

Does anybody in the family suffer from **longstanding lower back pain** ? YES/NO

If yes, what is the relationship to you? **_______________________
**

**
Thank you for taking your time to fill in this questionnaire!
**

## References

[1] Khan MA. Update on spondyloarthropathies. Ann Intern Med 2002;136:896–907. 12069564 10.7326/0003-4819-136-12-200206180-00011

[2] Dougados M van der Linden S Juhlin R Huitfeldt B Amor B Calin A The European Spondylarthropathy Study Group preliminary criteria for the classification of spondylarthropathy. Arthritis Rheum 1991;34:1218–27. 1930310 10.1002/art.1780341003

[3] Dougados M van der Heijde D. Ankylosing spondylitis: how should the disease be assessed? Best practice & research. Clin Rheumatol 2002;16:605–18. 10.1053/berh.2002.0252 12406429

[4] Ehrenfeld M. Geoepidemiology: the environment and spondyloarthropathies. Autoimmun Rev 2010;9:A325–9. 20026258 10.1016/j.autrev.2009.11.012

[5] Haroon NN Paterson JM Li P Haroon N. Increasing proportion of female patients with ankylosing spondylitis: a population-based study of trends in the incidence and prevalence of AS. BMJ Open 2014;4:e006634. 10.1136/bmjopen-2014-006634 PMC4267076 25510888

[6] Dougados M Hochberg MC. Why is the concept of spondyloarthropathies important? Best Pract Res Clin Rheumatol 2002;16:495–505. 12406423

[7] Gonzalez-Roces S Alvarez MV Gonzalez S Dieye A Makni H Woodfield DG HLA-B27 polymorphism and worldwide susceptibility to ankylosing spondylitis. Tissue Antigens 1997;49:116–23. 9062966 10.1111/j.1399-0039.1997.tb02724.x

[8] Londoner. The world in one city. 1 2004, page 4. www.london.gov.uk.

[9] Calin A Porta J Fries JF Schurman DJ. Clinical history as a screening test for ankylosing spondylitis. JAMA 1977;237:2613–4. 140252

[10] Rudvaleit M van der Heijde Landewe R Listing J Akkoc N Brandt J The development of assessment of spondyloarthritis international society (ASAS) classification criteria for Axial Spodyloarthritis (part II). Validation and final selection. Ann Rheum Dis 2009;(68):777–83. 19297344 10.1136/ard.2009.108233

[11] Rudvaleit M van der Heijde Landewe R Akkoc N Brandt J Chou CT The Assessment of SpondyloArthritis International Society (ASAS) classification criteria for peripheral Spodyloarthritis and Spondyloarthritis in general. Ann Rheum Dis 2011;70:25–31. 21109520 10.1136/ard.2010.133645

[12] Roussou E Jurik AG. Difficulties for the detection of positive signs of sacroiliitis in spondyloarthritides by magnetic resonance imaging (MRI) in everyday clinical practice. Clin Exp Rheumatol 2011;29(3)594–5. 21722504

[13] Garrett S Jenkinson T Kennedy LG Whitelock H Gaisford P Calin A. A new approach to defining disease status in ankylosing spondylitis: The Bath Ankylosing Spondylitis Disease Activity Index. J Rheumatol 1994;21:2286–91. 7699630

[14] Calin A Garrett S Whitelock H Kennedy LG O’Hea J Mallorie P A new approach to defining functional ability in ankylosing spondylitis: The development of the Bath Ankylosing Spondylitis Functional Index. J. Rheumatol 1994; 21:2281–5. 7699629

[15] Reveille JD. The genetic basis of spondyloarthritis. Ann Rheum Dis 2011;70(Suppl 1):i44–50 21339218 10.1136/ard.2010.140574

[16] Reveille JD Ball EJ Khan MA. HLA-B27 and genetic predis-posing factors in spondyloarthropathies. Curr Opin Rheumatol 2001;13:265–72. 11555726 10.1097/00002281-200107000-00004

[17] Ciurtin C Roussou E. Cross-sectional study assessing family members of psoriatic arthritis patients affected by the same disease: differences between Caucasian, South Asian and Afro-Caribbean populations living in the same geographic region. Int J Rheum Dis 2013 8;16(4):418–24. 23992262 10.1111/1756-185X.12109

[18] Hartshorne ST. Dermatological disorders in Johannesburg, South Africa. Clin Exp Derm.2003;6:661–5. 10.1046/j.1365-2230.2003.01417.x 14616837

[19] Zhaos S Challoner B Khattak M Moots R Goodson NJ. Increasing smoking intensity is associated with increased disease activity in axial SpA. Rheum Int 2017;37(2)239–44. 10.1007/s00296-016-3590-4 PMC5258786 27815702

[20] Burch VC Isaacs S Kalla AA Ethnicity and patterns of spondyloarthritis in South Africa-analysis of 100 patients. J Rheumatol 01 10 1999;26(10):2195–200. 10529139

[21] Mijiyawa M Oniankitan O Khan MA. Spondyloarthropathies in sub-Saharan Africa. Curr Opin Rheumatol 2000 7;12(4):281–6. 10910180 10.1097/00002281-200007000-00008

[22] Adizie T Moots RJ Hodkinson B French N Adebajo AO. Inflammatory arthritis in HIV positive patients: A practical guide. BMC Infect Dis 2016;16:100. 26932524 10.1186/s12879-016-1389-2 PMC4774153

